# Supplementary material for: A Celsr3 Mutation Linked to Tourette Disorder Disrupts Cortical Dendritic Patterning and Striatal Cholinergic Interneuron Excitability
Source: Int J Mol Sci. 2025 Oct 23;26(21):10307. doi: 10.3390/ijms262110307 (PMC12610430; doi:10.3390/ijms262110307)
Supplement: Supplementary file 1 [file ijms-26-10307-s001.zip › ijms-3849933-supplementary.pdf]

Supplemental Materials

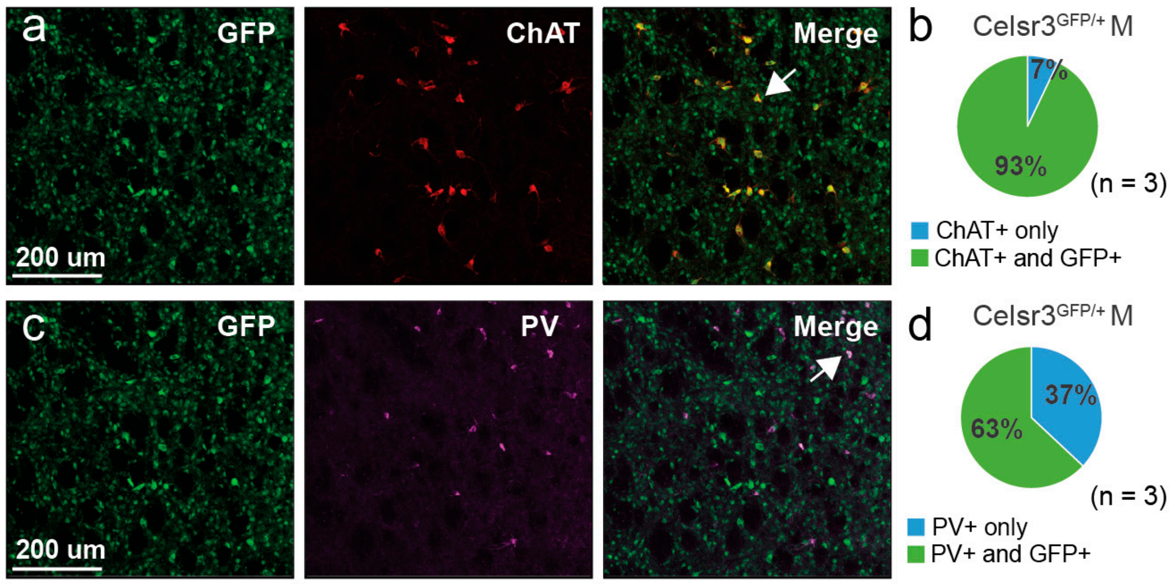

Supplementary Figure S1. | Striatal cholinergic interneurons show more colocalization with Celsr3 than striatal PV interneurons. **(a)** Representative images from a *Celsr3*$^{eGFP/+}$ reporter mouse taken from central striatal ROIs (50 um thickness), ChAT+ cells. **(b)** Percentage of colocalized GFP+ and ChAT+ cells (n = 254 cells, n = 8 slices, n = 3 mice) in the dorsal striatum from (A/P range ~1.2 - 0.4 from bregma). 93% of ChAT+ cells express *Celsr3* (GFP+). **(c)** Representative images from a *Celsr3*$^{eGFP/+}$ reporter mouse taken from central striatal ROIs (50 um thickness), PV+ cells. **(d)** Percentage of colocalized GFP+ and PV+ cells (n = 238 cells, n = 8 slices, n = 3 mice) in the dorsal striatum from (A/P range ~1.2 - 0.4 from bregma). 63% of PV+ cells express *Celsr3*.

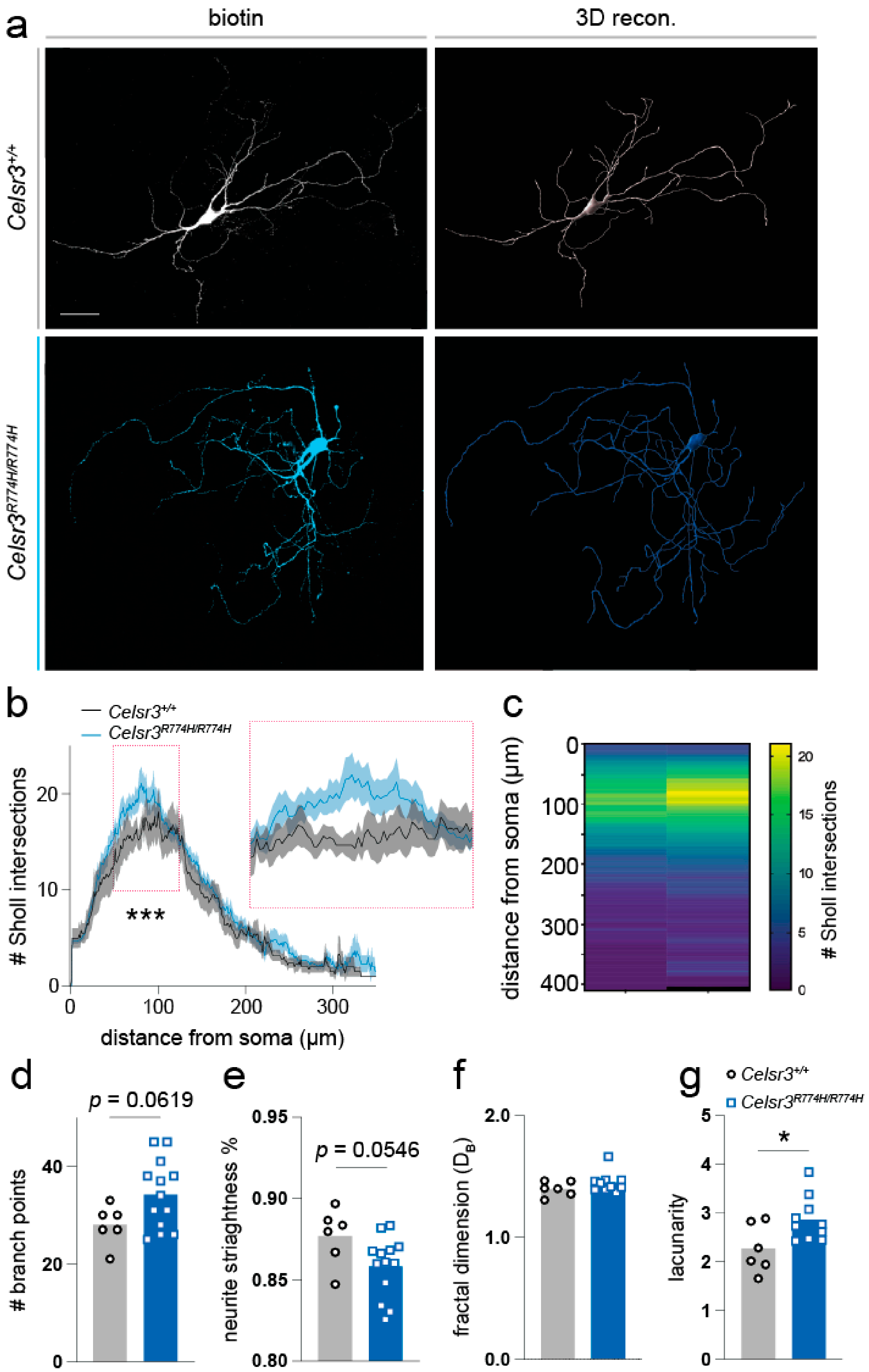

a
biotin
3D recon.
Celsr3+/+
Celsr3R774H/R774H
b
Celsr3+/+
Celsr3R774H/R774H
Sholl intersections
***
distance from soma (μm)
c
distance from soma (μm)
Sholl intersections
d
p = 0.0619
# branch points
e
p = 0.0546
neurite striaghtness %
f
fractal dimension (DB)
g
Celsr3+/+
Celsr3R774H/R774H
*
lacunarity

Supplementary Figure S2. | **Increased neurite complexity and altered spine-like protrusions in striatal cholinergic interneurons of *Celsr3*^{R774H}-mutant mice**. **(a)** Representative images of confocal maximum intensity projections of biotin-filled neurons (left) and their 3D reconstructions (right) in *Celsr3*$^{+/+}$ (top) and *Celsr3*$^{R774H/R774H}$ (bottom) mice. Scale bar represents 50 µm. **(b)** Sholl analysis of *Celsr3*$^{+/+}$ (n = 6) and *Celsr3*$^{R774H/R774H}$ (n = 13) reconstructed neurons (genotype effect: p < 0.001, 2way ANOVA). Inset shows enlargement of Sholl plot ROI (magenta dotted box). **(c)** Heat map of Sholl intersections vs. distance from soma in *Celsr3*$^{+/+}$ (left) and *Celsr3*$^{R774H/R774H}$ mice (right). **(d)** Total number of branch points (p = 0.0619, t-test). **(e)** Neurite straightness score (p = 0.0546, t-test). **(f)** Fractal dimension (p =0.2635, Mann-Whitney test, left) and **(g)** lacunarity (p = 0.0379, t-test) measures. 3 neurons were excluded from the *Celsr3*$^{R774H/R774H}$ group for this analysis due to background pixels that interfered with Db and lacunarity scoring. $p < .05$ (*), $p < .01$ (**), $p < .001$ (***).

Supplemental Table S.1

Table S1. Mouse lines.

Line Name	Description/Use	Supplier	Stock#
A2a-Cre	Cre recombinase expressed under control of *A2a*, used to visualize indirect pathway axons in mouse brain	MMRRC	036158-UCD
Ai14	Reporter line that expresses TdTomato in Cre recombinase expressing cells, used to visualize direct and indirect pathway axons when crossed with Drd1-Cre and A2a-Cre, respectively, and used to quantify density and nearest neighbor distribution of cortical and striatal SSTINs	JAX Mice	007914
C57BL/6	Wild type inbred line used as a background strain, and for backcrossing + line refreshing	JAX Mice	000664
Celsr3-eGFP	Knock-in eGFP line used to study *Celsr3* expression patterns in mouse brain	Mario Cappechi, University of Utah [47]	RRID:MGI: 3849330
Celsr3^{R774H}	Line carrying point mutation in *Celsr3*, used in all experiments	generated in house	n/a
Chat-eGFP	BAC transgenic line expressing eGFP in cholinergic cells, used to quantify density of striatal Cholinergic interneurons + for targeted recordings in dorsolateral striatum	JAX Mice	007902
Drd1-Cre	Cre recombinase expressed under control of *Drd1*, used to visualize direct pathway axons in mouse brain	MMRRC	030989-UCD
Pvalb-Cre	Cre recombinase expressed under control of *Pvalb*, used for off-target sparse cell labelling of layer V cortical neurons	JAX Mice	012358
Sst-Cre	Cre recombinase expressed under control of *Sst*, used to quantify cortical + striatal SSTIN density	JAX Mice	028864

Supplemental Table S.2

Table S2. Criteria for spine classification.

Spine Class	Criteria
Stubby	Spine length < 1 μm
Mushroom	Spine length < 3 μm and spine head width > spine neck width*2
Long Thin	Spine head width ≥ spine neck width
Filopodia	True
